# The mediating role of depressive symptoms among Turkish population related to gender and low back pain: evidence from a national health survey

**DOI:** 10.1186/s12889-024-18612-9

**Published:** 2024-04-23

**Authors:** Nadire Gülçin Yıldız, Halide Z. Aydin, Grace Sambo, Kemal Aydın, Hatice Yıldız, Ichtiarini Nurullita Santri, Yuniar Wardani, Bwanalori Mwamulima, Khoiriyah Isni, Yohane V. A. Phiri

**Affiliations:** 1 Faculty of Education, Department of Guidance and Counseling, Istanbul Medipol University, Istanbul, Turkey; 2 Arnold School of Public Health, University of South Carolina, Columbia, SC USA; 3 Chang Gung Medical Education Research Centre (CG-MERC), Chang Gung Memorial Hospital, Taoyuan, Taiwan; 4 Faculty of Economics and Administrative Sciences, Amasya University, Amasya, Turkey; 5 Health Sciences Institute, Istanbul Medipol University, Istanbul, Turkey; 6 Faculty of Public Health, Universitas Ahmad Dahlan, Yogyakarta, Indonesia; 7 Directorate of Health and Social Services, Rumphi District Council, Rumphi, Malawi; 8 Department of Epidemiology and Environmental Health (EEH), University at Buffalo, Buffalo, NY USA; 9 Charis Professional and Academic Research Consultants (CPARC), C/O, Mchinji, P.O. Box 132, Malawi

**Keywords:** Low back pain, Depression, Sociodemographic factors, Behavior-related factors, Türkiye health survey

## Abstract

**Background:**

Low back pain (LBP), though non-life-threatening, burdens healthcare with treatment expenses and work hours lost. Globally, 70–84% experience it, with risk factors tied to societal structure, income, and living conditions, making it a leading cause of disability.

**Methods:**

This study utilized data from the 2019 Türkiye Health Survey, which consisted of 17,084 individuals aged 15 and above. Our study focused on investigating the factors related to low back pain through a cross-sectional analysis. To analyze these factors, we employed binary multivariate logistic regression. Additionally, we conducted post-hoc analyses to assess the potential mediating effect of depressive symptoms on the relationship between low back pain and gender.

**Results:**

We found that 31.9% of the population experienced low back pain, with women being 58% more likely [aOR = 1.58; 95% CI (1.45–1.73)] than men to report symptoms. Individuals aged 55 + years old had a 90% [aOR = 1.90; 95% CI (1.61–2.23)] chance of experiencing low back pain, indicating an age-related increase. In the general population, having depressive symptoms was 2.49 [95% CI (2.23–2.78)] times more likely associated with low back pain. Our mediation analysis showed that gender (i.e., women vs. men), indicated by direct effects with β-estimates e = 0.78, predicted the likelihood of low back pain. Additionally, the relationship between gender and low back pain, mediated through a history of depressive symptoms, had a significant total indirect effect (i.e., β-estimate given as e = 0.49). Specifically, a history of depressive symptoms accounted for 17.86% [95% CI (9.67–20.10)] of the association between women having a higher likelihood of low back pain compared to men.

**Conclusion:**

We observed that a higher likelihood of low back pain associated with gender and aging. Additionally, BMI served as a significant predictor, particularly in adults. Depression mediated the association between gender and low back pain. Acknowledging these associations may help identify and address contributing factors to LBP, potentially increasing awareness and alleviating the burden. Policymakers and healthcare professionals may consider these findings when developing prevention and treatment programs for low back pain.

## Introduction

Low back pain (LBP) is a musculoskeletal disorder that is prevalent worldwide. It has a profound impact on individuals in all professions, including both the business world and the general population. LBP causes pain, reduces productivity, lowers the quality of life, and increases medical costs [1, 2]. Although LBP is not life-threatening, it imposes a significant health burden due to the cost of treatment and loss of work hours [3]. LBP is one of the primary causes of disability worldwide, and approximately 70–84% of the global population is known to experience LBP during their lifetime [4].

The risk factors for lower back pain may differ depending on the societal structure, income level, and living conditions [5, 6]. Epidemiological studies have found that an individual’s socioeconomic and occupational status, as well as personal traits such as exercise, alcohol use, and age, are crucial determinants of lower back pain [7–9]. Research conducted worldwide has indicated that depression and anxiety [10, 11], sedentary lifestyles, and obesity [12, 13] are some of the most common individual-level risk factors associated with lower back pain. However, an individual’s education level is believed to play an essential role in adapting to treatment and learning how to protect oneself from risk factors [14, 15]. Educational level is therefore considered a protective factor for lower back pain.

The evidence that exists shows that there are gender differences in populations with a history of low back pain, with women having a higher prevalence than men [16, 17]. Female sex hormones have been linked to playing a significant role in the development and progression of various musculoskeletal and degenerative diseases [16]. Pregnancy, childbearing, the physical and emotional stress of raising children, and weight gain during perimenopause have also been connected to low back pain in women [16, 18]. Postmenopausal women often experience accelerated disc degeneration due to a deficiency in estrogen. However, men are also at a risk of LBP due to their involvement in high-impact physical activities and certain lifestyle habits such as smoking, occupational factors, ergonomic factors, and chronic illnesses [16, 17]. It is worth noting that regardless of gender, certain important lifestyle habits are associated with chronic low back pain symptoms. Alcohol consumption and cigarette smoking are two lifestyle factors linked to low back pain. Drinking alcohol while experiencing low back pain can lead to resistance to medication and limit the pain-relieving effects, thus worsening the condition [18]. Chronic cigarette smoking, unlike alcohol consumption, alters pain perception and is associated with a higher level of pain intensity [18, 19].

Depression is likely to occur among individuals with symptoms of, and it is strongly linked to a high probability of disability [20]. Although depression and LBP can occur separately, they are also comorbid, and there are noticeable differences in how often they occur based on sex [21].. It has been hypothesized that women are more susceptible to experiencing depressive symptoms compared to men [22, 23]. This is believed to be due to their increased likelihood of facing chronic negative circumstances, having a low sense of mastery, and being less likely to engage in ruminative coping [22–24]. However, it is also argued that rumination alone does not fully explain the differences in depression between genders [24, 25]. Instead, the association between rumination and depression is stronger, suggesting that targeting rumination through intervention could potentially reduce the incidence of major depressive disorders. The vulnerability-stress approach model explains the gender differences in depressive symptom presentation, considering the affective, biological, and cognitive (ABC) vulnerabilities [24]. However, this model alone is insufficient in explaining the sex differences in depressive symptoms, as other psychosocial factors also play a significant role in their development [24]. Overall, depression and low back pain often occur together and can exacerbate each other. Previous studies have demonstrated that depression serves as a mediator in the relationship between sex and the occurrence of low back pain or pain incidence [26]. This indicates that the association between gender and low back pain, mediated by depression, involves a complex interplay of biological, psychological, and social factors.

Epidemiological studies conducted in Türkiye using individual-level samples have revealed that the annual prevalence of LBP ranges from 35 to 46%, while chronic LBP is prevalent between 13% and 18% [27–29]. These studies have also shown that women are twice as likely as men to experience LBP in their lifetime. Depending on the severity and duration of LBP, it can hinder daily activities and reduce the quality of life, which can lead to depression and anxiety [5]. Frequent hospital visits, sick leave, and early retirement have been associated with an increase in the prevalence of LBP in Turkish adults [29]. Predictors of LBP include a history of depressive and anxiety symptoms and an increase in body mass index (BMI) [30, 31]. However, some studies have found no significant association between hypertension and LBP [32]. The bidirectional results of these studies highlight the need for further research into the determinants of LBP in adult populations.

Studies exploring the determinants of LBP in Türkiye employing national representative samples are limited. Previously studies that have been conducted in Türkiye amongst individuals with LBP problems are either within a local setting or at a clinical level [13, 28, 29]. Dating back 2008, a standardized survey has been carried out in 27 European countries, the United Kingdom, Norway, Iceland and Türkiye [33, 34]. This survey is called the European Health Interview Survey (EHIS) in the European Countries and Türkiye Health Survey (THS) in Türkiye. In Europe, countries like Spain [35] have employed the EHIS to analyze the association between LBP and its determinants. This is unlike in Türkiye where the data on LBP in the THS has not been fully utilized to explicate the association between LBP and various determinants. However, it is important to note that research findings differ across countries, and an increasing number of studies indicate that sociodemographic, behavioral factors, and medical conditions are linked to lower back pain [8, 36, 37]. Therefore, using 2019 THS national representative microdata, this study aimed to examine the association between LBP and demographic, socioeconomic and behavioral factors. Additionally, we performed a post hoc mediation analysis using depressive symptoms as the mediator to examine the association between gender and LBP.

## Materials and methods

### Study design and setting

In this cross-sectional study, the 2019 Türkiye Health Survey (THS) questionnaire was designed to collect information on socio-demographic data, health status, health determinants, access to healthcare services, and associated factors. For this study, we utilized self-reported data from all participants, which included information on their socio-demographic characteristics (such as age, marital status, education, physical activity level, and BMI status), any diseases or conditions experienced in the past 12 months (i.e., low back pain), and the level of depressive symptoms experienced in the past two weeks as assessed by the Patient Health Questionnaire-8 (PHQ-8). All participants enrolled into the study either individually or their parents/guardians, for those aged less than 18 years old, signed a written consent to participate in the study. Access to anonymized microdata was provided by the Türkiye Statistical Institute under an agreement that outlined the security, confidentiality, accessibility, and appropriate use of the data.

### Participants

The 2019 THS had a total of 23,199 participants in the entire population, with 17,084 individuals being 15 years old and above. For our analysis, we selected the 17,084 individuals of both genders aged 15 years and older who had complete data on sociodemographic characteristics, PHQ-8 questionnaire (depressive symptoms assessment) and LBP. We aimed to conduct a population-based study, which is why we included all participants aged 15 years and older with complete data. Trained personnel from the Türkiye’s Statistical Institute conducted face-to-face interviews with individuals from each selected household using computer-assisted methods. A complete description of the sampling methodology can be found elsewhere [38].

### Study measurements

#### Outcome measure

The outcome variable was current status of low back pain, low back pain disorder and any other chronic back defects among adults. THS gathers data on the annual prevalence of chronic diseases. The question for all chronic diseases were dichotomously coded ‘Yes’ or ‘No’ for presence and absence of history of the disease condition, respectively. Similarly, to assess LBP amongst adults, each of the participants was asked, “during the past 12 months, have you had low back pain, low back disorder or other chronic back defects?”.

#### Independent variables

Based on literature we selected a number of independent variables including sociodemographic characteristics such as gender (male or female), age (15–34, 35–54, and 55+), marital status (single, married, widowed and divorced), education level (primary level and below, secondary level and equivalent, high school level and equivalent, university level and above), work status (employed, housework, job seeker, continuing education, and retired/disabled) and birth place (rural or urban) [12]. Additionally, we considered individual level characteristics including depressive symptoms (no or yes), body mass index (BMI) (normal weight, pre-obese, obese), and physical activity (no activity/mild activity, moderate activity, intense activity) [12, 30].

#### Patient health questionnaire (PHQ-8) tool

Of interest THS includes question used to assess depressive symptoms amongst adults employing the patient health questionnaire (PHQ-8) tool. The PHQ-8 module consists of eight Likert-type items and measures the frequency of exposure to depression symptoms in the last two weeks. The PHQ-8 tool has the lowest score of zero and the highest of 24. The PHQ-8 is a validated tool that has been used in both Turkey [39, 40] and other countries [41], with tested measures of reliability. For our study, assessment of depression was calculated employing the algorithm where scores of 5–9, 10–14 and 15–24 were accordingly classified as mild, moderate, moderate to severe depressive symptoms. In general, the cut of point score was 10, where participants with PHQ-8 greater than 10 were defined as having reported depressive symptoms. We, hence, coded a binary variable with ‘YES’ for those with scores greater than 10 and ‘NO’ for those less than 10.

#### The international physical activity questionnaire (IPAQ) module

Additionally, THS used the International Physical Activity Questionnaire (IPAQ) module which consists of eight items to record how much time each of the participant spent on walking, cycling, sports, fitness, and muscle-strengthening activities each day. The IPAQ is a standardized questionnaire used to assess the intensity and duration of physical activity and sedentary behavior in individuals’ daily routines. These measurements are then analyzed to estimate the total amount of physical activity in metabolic equivalent of task (MET) in minutes per week and the time spent sitting. The tool’s validity and reliability have been extensively assessed, and it is widely used worldwide [42–44]. The Turkish version of IPAQ has also been validated [45]. According IPAQ measurement criteria, walking less than ten minutes was excluded from calculation of total exercise time which is expressed in minutes and multiplied by metabolic equivalents (MET) values. In our study, participants with MET values below 600 per week for walking, cycling, sports, fitness, and muscle strengthening were classified inactive. Similarly, participants with MET values of equal to 600, up to 3000 and above 3000 were classified as minimally active, moderately active, and highly active respectively.

### Statistical analysis

We employed Chi-square test to examine the distribution of both sociodemographic and other characteristics between those with and without LBP. The association between the outcome and independent variables was analyzed employing binary logistic regression. All the variables included in our analyses as our independent variables were selected based on their importance in literature [12, 46]. All variables with a *p*-value of < 0.1 were selected for inclusion in multivariable models [47]. To assess multicollinearity of the variables included in in the final model, variance inflation factor (VIF) and tolerance were used with VIF < 10 and tolerance > 0.1 indicating no multicollinearity problems in our models. To assess the goodness of fit of our independent variables as predictors of LBP, Hosmer-Lemeshow test (HL test) was employed [31]. We conducted a post-hoc mediation analysis using CAUSALMED procedure in SAS [48] to examine the relationship between gender (X) and LBP (Y), using depression as the mediator (M). The CAUSALMED procedure calculates a three-way breakdown of causal mediation analysis (CMA), which divides the total effect of a treatment into direct and indirect effects. The indirect effect is conveyed to the outcome through a mediator (M) and we tested the significance of the indirect effect through the mediator (M) by employing a bootstrapping statistics [49]. Bootstrapping entails extracting samples from the data set multiple times and estimating the indirect effect in each resampled data set. This process is repeated numerous times to create an empirical estimate of the sampling distribution of the indirect effect. This estimate is then utilized to generate confidence intervals. To generate bootstrap confidence intervals (90%, 95%, and 99%) for the indirect effects, we utilized 1000 bootstrap samples. The results for our final models were reported as adjusted odds ratios (AOR) and their 95% confidence intervals with the statistical power set at *p* < 0.05. All statistical analyses were performed using SAS 9.4 (SAS Institute Inc., Cary, NC, USA).

### Ethical approval and consent to participate

The THS study protocol received approval from the chairperson of the Türkiye Statistical Institute, following the “Regulation on Procedures and Principles Regarding Confidential Data Privacy and Data Security in the Official Statistics,” to maintain data confidentiality. The guidelines were officially published and gazetted on 20/06/2006, with reference number 26,204. The experimental protocols used to collect data from participants in the study were approved by the ethics committee of the Türkiye Statistical Institute. Prior to their inclusion in the survey, the study participants or their guardians provided informed and written consent. Our study methods adhered to the ethical guidelines and regulations outlined in the authorized ethical approval and consent guidelines.

## Results

### Prevalence of low back pain

**Fig. 1:**
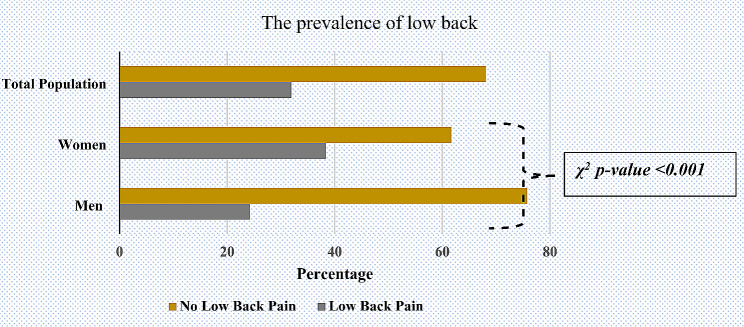
The prevalence of low back pain in the Turkish population and its distribution by gender. * χ-^*2*^*Chi-Square tests, bold means significant i.e., p < 0.05*

We recruited a total of 17,084 participants aged 15 years and older. Figure 1 represents the prevalence of LBP by gender amongst our study participants. Women had a prevalence rate of 38.31%, which was almost twice as high as men’s rate of 24.24%. The population prevalence was over half that of women, at 31.91%.

In Table 1, we further observed that with increase in age, the prevalence of participants who reported having experienced LBP within the past 12 months equally increased. Those who were married (75.66%), had secondary education and equivalent (45.07%), retired/disabled (51.40%) and were rural based (97.60%) had a higher prevalence of LBP. We also observed that individuals who reported having depressive symptoms (17.37%, *n* = 947) and a normal BMI status (91.50%, *n* = 4949), as well as reporting no or mild physical activity (66.76%, *n* = 3639), reported experiencing lower back pain. There were notable differences observed in the sociodemographic characteristics between individuals who experienced low back pain and those who did not (*p* < 0.05), except for the variable of birthplace (*p* < 0.085).

**Table 1:** Distribution of individual and sociodemographic variables by low back pain status

Variable	Low back pain				
	NO		YES		*p-value* ^*c*^
	n	%	n	%	
**Gender**					**< 0.0001**
*Male*	5896	50.68	1888	34.64	
*Female*	5737	49.32	3563	65.36	
**Age Group**					**< 0.0001**
*15–34*	4867	41.84	933	17.12	
*35–54*	4068	34.97	2245	41.19	
*55+*	2698	23.19	2273	41.7	
**Marital Status**					**< 0.0001**
*Single*	3150	27.08	460	8.44	
*Married*	7602	65.35	4124	75.66	
*Widowed*	368	3.16	206	3.78	
*Divorced*	513	4.41	661	12.13	
**Educational Level**					**< 0.0001**
*Primary level and below*	1079	9.28	1115	20.45	
*Secondary level and equivalent*	4275	36.75	2457	45.07	
*High School level and equivalent*	3872	33.28	1219	22.36	
*University and above*	2407	78.48	660	21.52	
**Work Status**					**< 0.0001**
*Employed*	3683	31.66	1217	22.33	
*Housework (Not working)*	1113	9.57	514	9.43	
*Job seeker*	1195	10.27	108	1.98	
*Continuing education*	1449	12.46	810	14.86	
*Retired / Disabled*	4193	36.04	2802	51.4	
**Birth Place**					0.085
*Rural*	11,300	97.14	5320	97.6	
*Urban*	333	2.86	131	2.4	
**Depressive symptoms**					**< 0.0001**
*Yes*	754	6.48	947	17.37	
*No*	10,879	93.52	4504	82.63	
**Body Mass Index**					**< 0.0001**
*Normal weight*	11,137	96.12	4949	91.5	
*Pre-obese*	350	3.02	340	6.29	
*obese*	99	0.85	120	2.22	
**Physical Activity**					**< 0.0001**
*No/Mild activity*	6474	55.65	3639	66.76	
*Moderate activity*	2724	23.42	1026	18.82	
*Intense activity*	2435	20.93	786	14.42	

^*c*^ *p-value from Chi-Square tests, bold means significant i.e., p < 0.05*

In this study, we conducted a further investigation into the distribution of different variables based on age groups. According to Table 2, a greater number of women were recruited for the study across all three age groups (15–34, 35–54, and 55+). We also found that individuals who were married (*n* = 5500), had attained a secondary school education or equivalent (*n* = 2768), and were born in rural areas (*n* = 6132) constituted the highest categories within the age group of 35–54 years. The age group of 35–54 years also had the highest percentage of participants who experienced depression (*n* = 743), followed by those aged 55 + years (*n* = 601). Additionally, we observed that participants within the age group of 55 + years had a higher prevalence of lower back pain (*n* = 2273), while those within the age group of 35–54 years had a slightly lower prevalence (*n* = 2245).

**Table 2:** Distribution of individual and sociodemographic variables by age groups

Variables	Age of the participants						*p*-value^c^
	15–34 Years		35–54 Years		55 + Years		
	n	%	n	%	n	%	
**Gender**							
*Male*	2671	46.05	2855	45.22	2258	45.42	0.6409
*Female*	3129	53.95	3458	54.78	2713	54.58	
**Marital Status**							
*Single*	3174	54.72	354	5.61	82	1.65	**< 0.0001**
*Married*	2532	43.66	5500	87.12	3694	74.31	
*Widowed*	89	1.53	319	5.05	166	3.34	
*Divorced*	5	0.09	140	2.22	1029	20.7	
**Educational Level**							
*Primary level and below*	271	4.67	513	8.13	1410	28.36	**< 0.0001**
*Secondary level and equivalent*	1594	27.48	2768	43.85	2370	47.68	
*High School level and equivalent*	2398	41.34	1909	30.24	784	15.77	
*University and above*	1537	26.5	1123	17.79	407	8.19	
**Work Status**							
*Employed*	2054	26.05	2536	40.17	310	10.75	**< 0.0001**
*Housework (Not working)*	333	4.22	832	13.18	462	16.01	
*Job seeker*	1300	16.48	3	0.05	0	0.00	
*Continuing education*	1957	24.82	302	4.78	0	0.00	
*Retired / Disabled*	2242	28.43	2640	41.82	2113	73.24	
**Birth Place**							
*Rural*	5647	97.36	6132	97.13	4841	97.38	**< 0.0001**
*Urban*	153	2.64	181	2.87	130	2.62	
**Lower Back Pain**							**< 0.0001**
*Yes*	933	16.09	2245	35.56	2273	45.73	
*No*	4867	83.91	4068	64.44	2698	54.27	
**Depressive symptoms**							
*Yes*	357	6.16	743	11.77	601	12.09	**< 0.0001**
*No*	5443	93.84	5570	88.23	4370	87.91	
**Body Mass Index**							
*Normal weight*	5693	98.36	5885	93.81	4508	91.37	**< 0.0001**
*Pre-obese*	78	1.35	294	4.69	318	6.45	
*obese*	17	0.29	94	1.5	108	2.19	
**Physical Activity**							
*No/Mild activity*	3025	52.16	3694	58.51	3394	68.28	**< 0.0001**
*Moderate activity*	1368	23.59	1461	23.14	921	18.53	
*Intense activity*	1407	24.26	1158	18.34	656	13.2	

^***c***^ ***p-value from Chi-Square tests, bold means significant i.e., p < 0.05***

### The relationship between low back pain and sociodemographic and individual-level factors

Table 3 displays the results of the univariate analyses, which indicate that except for the birthplace of the participants, LBP was associated with all sociodemographic and individual-level factors. It is worth noting that among the entire population, experiencing LBP within the past 12 months was linked with reporting no/mild physical activity [rOR = 1.74; 95% CI (1.59–1.91)] and moderate physical activity [rOR = 1.17; 95% CI (1.05–1.30)]. Furthermore, men aged 35–54 years old who reported no physical activity [rOR = 1.47; 95% CI (1.29–1.67)] had a considerable higher odd of experiencing LBP compared to women [rOR = 1.43; 95% CI (1.25–1.66)]. These associations were only observed in the univariate analyses and not in the multivariate analyses.

**Table 3:** Univariate regression analysis for low back pain: Individual and sociodemographic variables*

Variables	Total Population		Gender				Age Group (Yrs.)					
			Men		Women		15–34		35–54		55+	
	rOR	95% CI	rOR	95% CI	rOR	95% CI	rOR	95% CI	rOR	95% CI	rOR	95% CI
**Gender (Ref = Male)**												
*Female*	**1.94**	**1.84–2.07**	**-**	**-**	**-**	**-**	**1.39**	**1.20–1.60**	**1.871**	**1.68–2.08**	**2.80**	**2.49–3.15**
**Age Group in Yrs. (ref = 15–34)**												
*35–54*	**2.88**	**2.64–3.14**	**2.42**	**2.11–2.78**	**3.27**	**2.92–3.66**	-	-	-	-	-	-
*55+*	**4.4**	**4.018–4.81**	**2.98**	**2.58–3.43**	**6.01**	**5.34–6.76**	-	-	-	-	-	-
**Marital Status (ref = single)**												
*Married*	**3.71**	**3.34–4.12**	**2.89**	**2.49–3.35**	**4.41**	**3.80–5.12**	**2.33**	**2.01–2.69**	**1.89**	**1.46–2.43**	1.30	0.83–2.05
*Widowed*	**3.83**	**3.15–466**	**2.30**	**1.59–3.34**	**4.41**	**3.46–5.63**	**2.20**	**1.31–3.69**	**2.09**	**1.49–2.92**	1.12	0.65–1.93
*Divorced*	**8.82**	**7.58–10.26**	**5.76**	**4.12–7.94**	**8.89**	**7.36–10.74**	-	-	**3.28**	**2.17–4.96**	**2.35**	**1.47–3.74**
**Educational Level (ref = primary)**												
*Secondary level and equivalent*	**0.56**	**0.51–0.61**	**0.67**	**0.54–0.83**	**0.66**	**0.59–0.74**	**0.72**	**0.53–0.97**	**0.74**	**0.61–0.90**	**0.61**	**0.53–0.69**
*High School level and equivalent*	**0.31**	**0.27–0.34**	**0.46**	**0.37–0.58**	**0.32**	**0.28–0.37**	**0.44**	**0.33–0.60**	**0.57**	**0.46–0.69**	**0.41**	**0.34–0.49**
*University and above*	**0.27**	**0.24–0.30**	**0.41**	**0.33–0.53**	**0.27**	**0.23–0.31**	**0.53**	**0.39–0.71**	**0.42**	**0.34–0.52**	**0.34**	**0.27–0.43**
**Work Status (ref = retired/disabled)**												
*Employed*	**0.49**	**0.46–0.54**	0.863	0.73–1.02	**0.55**	**0.49–0.62**	1.01	0.86–1.12	1.14	0.97–1.35	**1.66**	**1.22–2.27**
*Housework (Not working)*	**0.69**	**0.62–0.78**	0.966	0.79–1.18	**1.23**	**1.02–1.48**	1.04	0.78–1.40	1.18	0.11–3.11	**1.49**	**1.14–1.94**
*Job seeker*	**0.14**	**0.11–0.17**	**0.23**	**0.17–0.32**	**0.14**	**0.10–0.18**	**0.40**	**0.32–0.50**	1.15	0.89–1.49	-	-
*Continuing education*	**0.84**	**0.76–0.92**	**1.32**	**1.11–1.58**	**1.36**	**1.14–1.63**	1.05	0.92–1.16	**1.74**	**1.55–1.95**	-	-
**Birth Place (ref = urban)**												
*Rural*	1.20	0.98–1.47	0.95	0.69–1.32	0.75	0.58–0.98	0.83	0.52–1.32	0.765	0.55–1.06	0.90	0.63–1.27
**Depressive symptoms (ref = No)**												
*Yes*	**3.03**	**2.74–3.36**	**2.64**	**2.18–3.19**	**2.81**	**2.49–3.18**	**3.94**	**3.14–4.93**	**2.585**	**2.21–3.02**	**2.42**	**2.03–2.89**
**Body Mass Index (ref = normal weight)**												
*Pre-obese*	**2.19**	**1.88–2.55**	**2.10**	**1.59–2.78**	**1.97**	**1.64–2.38**	1.59	0.93–2.70	**1.35**	**1.07–1.71**	**2.11**	**1.67–2.66**
*obese*	**2.73**	**2.09–3.56**	**1.28**	**0.61–2.67**	**2.54**	**1.89–3.43**	**4.70**	**1.81–8.22**	1.26	0.83–1.90	**2.78**	**1.84–4.19**
**Physical Activity (ref = Intense activity)**												
*No/Mild activity*	**1.74**	**1.59–1.91**	**1.47**	**1.29–1.67**	**1.43**	**1.25–1.64**	**1.32**	**1.10–1.58**	**1.35**	**1.18–1.56**	**1.87**	**1.57–2.23**
*Moderate activity*	**1.17**	**1.05–1.30**	**1.24**	**1.07–1.44**	0.93	0.79–1.09	1.18	0.96–1.46	1.03	0.87–1.21	1.11	0.90–1.36

*rOR: relative Odds Ratios; 95% CI: 95% Confidence interval; Bold mean p < 0.05

Table 4 presents the results of our multivariate analyses, which revealed that women were 58% [aOR = 1.58; 95% CI (1.45–1.73)] more likely to report experiencing an episode of LBP compared to men. Additionally, the odds of experiencing LBP increased with age, with those aged 55 + years having a 90% [aOR = 1.90; 95% CI (1.61–2.23)] likelihood of LBP. Women aged 55 + years were 3.23 [95% CI (1.84–2.38)] times more likely to experience LBP compared to men of the same age, who had odds of 1.79 [95% CI (1.44–2.22)]. Participants who were divorced had a higher likelihood of reporting an episode of LBP in the general population [aOR = 2.03; 95% CI (1.68–2.46)]. Among those who were divorced, men ([aOR = 2.53; 95% CI (1.75–3.67)] had higher odds of reporting experiencing LBP compared to women [aOR = 2.01; 95% CI (1.58–2.55)].

**Table 4:** Multivariable association between low back pain and individual and sociodemographic variables*

Variables	Total Population		Gender				Age Group (Yrs)					
			Men		Women		15–34		35–54		55+	
	aOR	95% CI	aOR	95% CI	aOR	95% CI	aOR	95% CI	aOR	95% CI	aOR	95% CI
**Gender (Ref = Male)**												
*Female*	**1.58**	**1.45–1.73**	-	-	-	-	**1.29**	**1.08–1.53**	**1.53**	**1.34–1.75**	**1.90**	**1.61–2.23**
**Age Group in Yrs (ref = 15–34)**												
*35–54*	**1.92**	**1.73–2.12**	**1.59**	**1.34–1.88**	**2.09**	**1.84–2.38**	-	-	-	-	-	-
*55+*	**2.63**	**2.34–2.98**	**1.79**	**1.44–2.22**	**3.23**	**2.77–3.76**	-	-	-	-	-	-
**Marital Status (ref = single)**												
*Married*	**1.65**	**1.44–189**	**1.67**	**1.36–2.04**	**1.83**	**1.52–2.21**	**1.85**	**1.56–2.19**	**1.76**	**1.35–2.29**	1.40	0.87–2.24
*Widowed*	**1.45**	**1.16–1.81**	1.20	0.80–1.79	**1.70**	**1.29–2.24**	1.16	0.67–2.02	**1.75**	**1.24–2.45**	1.07	0.60–1.89
*Divorced*	**2.03**	**1.68–2.46**	**2.53**	**1.75–3.67**	**2.01**	**1.58–2.55**	-	-	**2.06**	**1.34–3.16**	1.58	0.97–2.56
**Educational Level (ref = primary)**												
*Secondary level and equivalent*	**0.80**	**0.71–0.89**	**0.77**	**0.61–0.98**	**0.84**	**0.74–0.95**	0.81	0.59–1.10	0.86	0.71–1.05	**0.83**	**0.72–0.97**
*High School level and equivalent*	**0.67**	**0.59–0.76**	**0.69**	**0.69–0.88**	**0.67**	**0.58–0.78**	**0.70**	**0.51–0.97**	**0.73**	**0.59–0.90**	**0.67**	**0.54–0.82**
*University and above*	**0.55**	**0.48–0.64**	**0.57**	**0.44–0.74**	**0.56**	**0.48–0.68**	**0.57**	**0.41–0.79**	**0.58**	**0.46–0.74**	**0.62**	**0.47–0.81**
**Work Status (ref = retired/disabled)**												
*Employed*	1.02	0.92–1.13	0.92	0.77–1.11	0.98	0.85–1.13	1.43	1.18–1.72	0.91	0.79–1.05	**1.58**	**1.43–1.78**
*Housework (Not working)*	0.99	0.87–1.13	0.83	0.67–1.02	1.25	1.023–1.53	1.32	0.96–1.81	0.94	0.78–1.13	**1.89**	**1.70–2.13**
*Job seeker*	**0.58**	**0.46–0.74**	**0.52**	**0.36–0.75**	**0.62**	**0.45–0.86**	**0.70**	**0.53–0.92**	1.11	0.10–2.62	**-**	**-**
*Continuing education*	**0.85**	**0.75–0.97**	0.85	0.69–1.05	0.98	0.81–1.20	0.86	0.78–1.13	0.91	0.69–1.19	**-**	**-**
**Depressive symptoms (ref = No)**												
*Yes*	**2.49**	**2.23–2.78**	**2.50**	**2.05–3.04**	**2.46**	**2.15–2.81**	**3.90**	**3.08–4.93**	**2.32**	**1.97–2.72**	**2.06**	**1.71–2.49**
**Body Mass Index (ref = normal weight)**												
*Pre-obese*	**1.40**	**1.19–1.65**	**1.78**	**1.33–2.37**	**1.23**	**1.01–1.50**	1.27	0.73–2.20	1.12	0.88–1.43	**1.68**	**1.31–2.14**
*obese*	**1.46**	**1.10–1.94**	1.01	0.47–2.17	**1.49**	**1.10–2.03**	**3.94**	**1.41–6.32**	0.89	0.58–1.36	**1.84**	**1.20–2.80**

*aOR: Adjusted Odds Ratios; 95% CI: 95% Confidence interval; Bold mean p < 0.05

The study’s findings indicated that regardless of gender and age group, education served as a protective factor against LBP. Those who completed secondary school, or an equivalent level of education were less likely to report experiencing LBP. While work status was protective of LBP symptoms in all subgroup analyses, this was not the case for individuals aged 55 years and above. The results revealed that being employed [aOR = 1.58; 95% CI (1.43–1.78)] or performing housework [aOR = 1.89; 95% CI (1.70–2.13)] were significantly associated with LBP among those aged 55 years and older compared to individuals who were retired or disabled.

In the general population, the study found that individuals with depressive symptoms had a 2.49 [95% CI (2.23–2.78)] times higher likelihood of reporting LBP. While women had a higher prevalence of both LBP and depressive symptoms, our findings indicated that men had a slightly greater chance of reporting LBP [aOR = 2.50; 95% CI (2.05–3.04)] compared to women [aOR = 2.46; 95% CI (2.15–2.81)]. Moreover, those aged 15–34 [aOR = 3.90; 95% CI (3.08–4.93)] and were depressed were more likely to report LBP than other age groups. Similarly, individuals in the same age group (15–34 years) who were obese were twice as likely as the overall population to report LBP [aOR = 3.94; 95% CI (1.41–6.32)].

### Depression as a partial mediator of the association between gender and low back pain

**Fig. 2:**
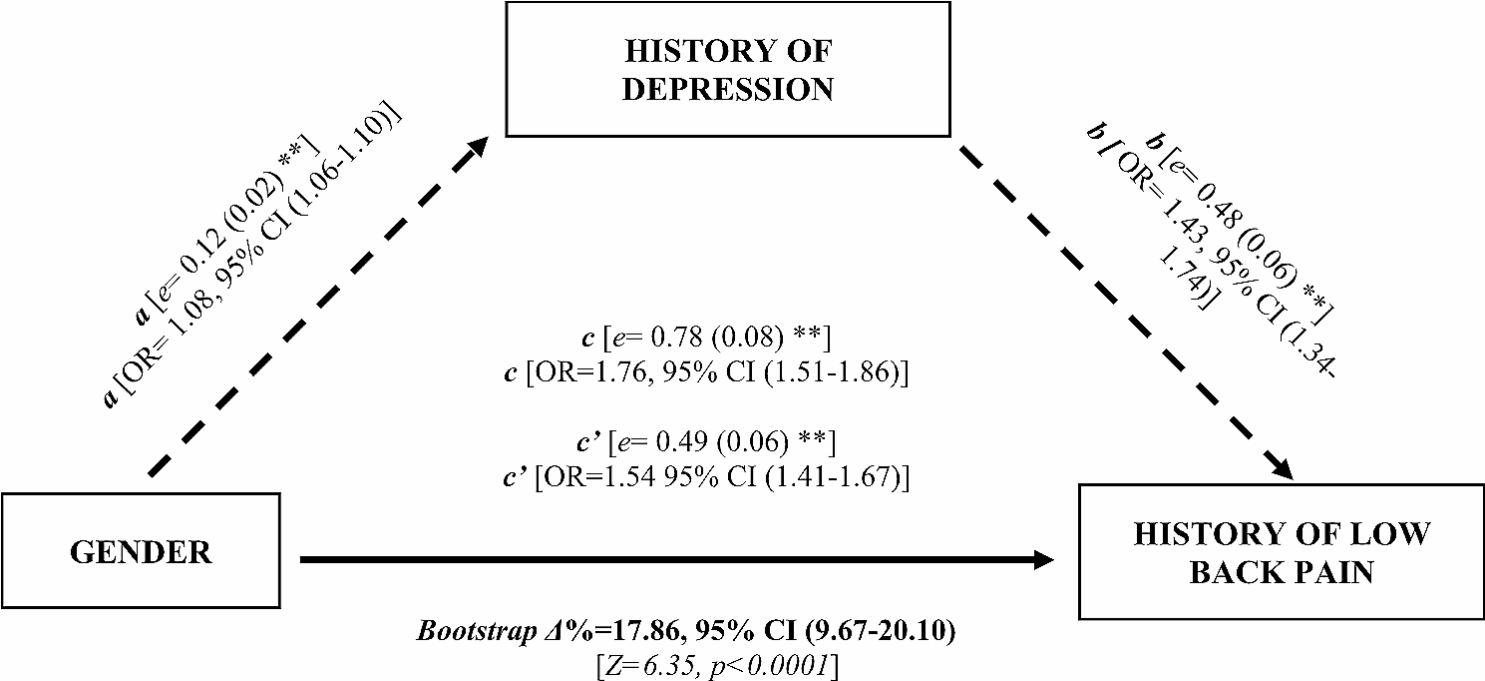
Mediation analysis with gender (Male or Female) as the predictor, history of depressive symptoms (defined as current status of depressive symptoms within the past 12 months, Yes/No) as the mediator, and the self-reported status of low back pain (LBP) (described as the current status of low back pain, low back pain disorder and any other chronic back defects within the past 12 months, with a binary measure) as the dependent variable. Depicted in the diagram (including *β-*effect estimates coefficients with their standard error) are all four requirements for a mediation effect which were satisfied: Path *a*, Path *b*, and Path *c* were significant. Path *c’* is significantly smaller than Path *c*. In detail, Path *a* represents the effect of gender on depressive symptoms. Path *b* represents the impact of depressive symptoms on low back pain (LBP) controlling for the gender effect. Together Path *a* and Path *b* represent the indirect (mediated) effect of gender on LBP through depressive symptoms. Path *c* represents the total effect of gender on LBP without the effect of the mediator. Path *c’* represents the direct effect of gender on LBP and is calculated controlling for the indirect (mediated) effect. The use of bootstrapping statistics indicated that Path *c’* was significantly smaller than Path *c* (*P* < 0.0001), which supports the fact that depression played a significant role as a mediator. *Where;a = path a.*. *b = path b.*. *c’ = direct effect, i.e., the effect of gender orientation on the current status of low back pain, mediated by history of depression (adjusted for age, education, marital status, work status and Body Mass Index).*. *c = effect of sex orientation on the current status of low back pain without the effect of the mediator.*. *e = β-effect estimates coefficients with standard error.*. *OR = Odds ratios of the effect.*. *95% CI = 95% Confidence Interval*. ^*⁎⁎*^*p < 0.001.*. *Δ%=Percentage change*

Based on the association we observed in the regression analyses, we conducted a mediation analysis between the independent variable gender (X), mediator depressive symptoms (M) and the dependent variable LBP (Y). The findings of the post hoc analyses are presented in Fig. 2 and show a partial mediation effect of depressive symptoms on the relationship between gender and low back pain (LBP). The results indicate that gender orientation had a significant effect (represented as e = Beta effect of estimates coefficients, standard errors in parathesis and ** signifying a p-value < 0.05) on depression (path *a* [*e* = 0.12 (0.02) **]) and depression on low back pain (path *b* [*e* = 0.48 (0.06) **]). In addition, the direct effect of gender on LBP was also significant (path *c* [*e* = 0.78 (0.08) **]). However, the study also found that the presence of depressive symptoms partially mediated the relationship between gender and LBP (c’ [*e* = 0.49 (0.06) **]), suggesting that depressive symptoms may explain some of the association between gender and LBP. The use of bootstrapping statistics further indicated that Path *c’* was significantly smaller than Path *c* [Δ%= 17.86%; 95% CI (9.67–20.10), Z = 6.5, *p* < 0.0001], which supports the fact that depression played a significant role as a mediator.

## Discussion

This study provides insights on the association between gender, low back pain (LBP), and depressive symptoms. The findings suggest that gender orientation determines the likelihood of presenting with LBP amongst the Turkish study population, however, the history of depressive symptoms plays a crucial role in partially mediating this association. According to the 2019 THS, the annual prevalence of LBP in Türkiye was 31.9%. Previous studies have indicated that the annual prevalence of LBP in adults ranges from 29 to 46% [50]. In the city of Afyon, Türkiye, the lifetime prevalence of LBP in adults aged 65 and older was found to be 51%, with chronic LBP at 13% [5]. LBP has a significant impact on the Turkish society, resulting in disability and frequent use of healthcare services [5, 50]. Our findings reveal that several sociodemographic and individual-level factors, such as gender, age, marital status, education level, employment status, obesity, and depression, are all associated with LBP.

We noticed that the participants’ age was a significant factor in predicting LBP, as the likelihood of experiencing LBP increased with age. The results of our study are consistent with previous research conducted in Türkiye. For example, a study carried out in the western province of Afyon found that individuals aged between 40 and 63 years had a higher likelihood of experiencing LBP than those aged between 20 and 39 years [5]. Similarly, another study conducted in Türkiye reported that adults over the age of 65 had a prevalence of LBP as high as 90% [50]. While LBP has been found to affect young adults between the ages of 20 and 40 years, the prevalence of LBP in adults is heightened by age-related medical conditions such as spine degeneration [51] and other physiological characteristics predetermined by gender [17, 51].

Our findings further showed that individuals between the ages of 35–54 who were divorced had a higher likelihood of experiencing LBP compared to those who were younger or older than them. To our knowledge, there is no direct relationship between divorce and LBP. However, divorce can be stressful, and it is well known that depression and anxiety are a risk factor for LBP [11, 52]. In addition, the emotional and physical strain of the divorce process may lead to changes in lifestyle (i.e., increased alcohol intake, reduced physical activity levels, and poor sleep quality) which may contribute to the development or exacerbation of LBP. Furthermore, LBP is prevalent in young and middle-aged adults and often labeled as ‘nonspecific’ and hard to identify [53]. The reason for the high prevalence of LBP in this age group can be attributed to the need to sustain a high level of daily activity, coinciding with the time when age-related changes start to occur in the lumbar spine and surrounding tissues, thus increasing the likelihood of experiencing LBP.

The results in our study further demonstrated that being a woman was associated with reporting for LBP. This finding is in line with several population-based studies which indicate that women have a higher risk of LBP than men attributable to a possible role played by hormonal influences [54]. There are various hypothesis explaining why women are at a higher odd of reporting of LBP due to hormonal influences. A systematic review employing 98 studies demonstrated that sex hormones in females play a vital role in the etiology of musculoskeletal degenerative conditions which may induce LBP signs and symptoms [17]. The higher risk of women experiencing or reporting of LBP can not only be explained by hormonal influences. A number of factors could be ascribed within a very complex interaction of sociodemographic factors and individuals level factors as a risk factor for reporting LBP in women [5]. The active involvement of women in formal employment is crucial for sustainable progress, social development, and prosperity of the any community [55]. However, in Türkiye, the percentage of women engaged in formal employment remains below 30%, with only 29.3% of women formally employed, while 70.7% are responsible for housework and domestic duties as housewives [55, 56]. The physically demanding nature of domestic work may significantly increase the risk of LBP in women [57]. Although, to our knowledge, no studies have investigated the possible association between domestic labor and LBP in the Turkish population. The fact that a significant proportion of women are domestic housewives in Türkiye makes it likely that the occurrence of LBP is higher in this population.

Current available evidence on the association between education and LBP, demonstrates that low formal education level is a risk factor for reporting LBP symptoms [37, 38]. There are a number of possible explanations to the observed association between low education level and LBP episodes. A systematic review of 68 papers suggest that the association is attributable to the influence of educational status on the variations in behavioral and environmental risk factors for LBP [37]. Furthermore, the study underscores the significance of differences in access and utilization of healthcare services, as well as adaptation to stressful events, as risk factors for LBP among individuals with low education levels compared to those with high education levels. These findings are consistent with the results of our study, where educational level was found to be protective against reporting LBP symptoms, regardless of gender and age. Similar results were also reported in Taiwan, where low education levels were associated with higher odds of LBP symptoms [58]. However, another study conducted in Taiwan suggested that a high level of education in certain occupations, such as nursing aides, is associated with higher odds of LBP [59].

Our analyses demonstrated that employment status had a bidirectional association with reported episodes of LBP. In the general population and gender subgroup analyses, there was a negative association between work status and LBP. However, in the age subgroup analyses, individuals who were 55 years or older and still employed or engaged in housework were found to have a greater likelihood of experiencing LBP. This could be because individuals aged 55 + years are already at a high risk of developing LBP due to the physiological changes that come with aging. Therefore, engaging in physically demanding tasks, whether formally or informally, is likely to lead to LBP symptoms [16, 60]. On the other hand, individuals who engaged in housework were found to have a greater likelihood of experiencing LBP. Several possible explanations for this finding have been previously documented. A study conducted in Japan among older individuals found that socioeconomic status, as measured by educational level, income, and occupational status, was a significant predictor of prolonged LBP [6]. In the study, the prevalence of LBP was highest among older adults who did not have a formal occupational status. Similarly, studies conducted in Türkiye have shown that LBP is a common condition reported by women who are housewives and list housework as their occupation [50, 61].

Obesity and low back pain (LBP) are two of the most common public health challenges in the general population. However, there is no clearly defined treatment for LBP, various methods of treatments have been shown to produce different outcomes within specific time frames [62]. One variable that has been believed to alleviate LBP is obesity management. Current literature on LBP treatment suggests that individuals who are pre-obese and obese should be advised to reduce their weight and exercise regularly [63, 64]. Our findings demonstrated that obesity was a significant predictor of LBP, and based on the results of our study, weight management recommendations for the treatment of LBP are plausible. However, a study conducted in the USA found no significant effect of obesity management in the treatment of LBP in both obese and non-obese individuals [63]. Similarly, a systematic review conducted by Chen et al. [64] revealed a lack of research on the effect of weight loss on LBP treatment. Our results, therefore, need to be carefully interpreted due to the cross-sectional nature of the study.

To our knowledge, this study is the first in Türkiye to investigate the role of depression as a partial mediator in the relationship between gender and LBP. Our findings indicate that depression partially mediates the link between gender and LBP. The debate on bidirectional association between depression and LBP is still ongoing [20, 21] and yet evidence also demonstrates that gender is associated with both LBP [5, 50] and depression [23, 24]. Conducted using a national representative survey, our study suggests that depression may play a significant role in explaining the gender differences observed in LBP. This is mainly because (1) gender differences appear to increase the likelihood of both LBP and depression, (2) depression is associated with LBP even after adjusting for gender, and (3) 17.86% of the effect of gender on LBP was indirectly mediated through depression. Gender roles and societal expectations can influence how men and women express emotional distress, with women being more likely to report depression and seek help, while men may suppress emotions, leading to somatic symptoms like low back pain [65]. Moreover, depression is often associated with increased stress, which can cause muscle tension and worsen low back pain [66]. Coping mechanisms used by individuals with depression, such as avoidance and withdrawal, may also contribute to the development or worsening of low back pain, particularly in men [67]. Importantly, research has established that depression is a contributing factor to chronic pain, including LBP, and that women are more prone to depression than men [10, 16, 20]. Depression can also affect pain perception and tolerance, as well as bodily inflammatory and immune responses, which can contribute to the onset and persistence of pain [68, 69]. We previously noted that women are more likely to experience depression and LBP due to biological, psychological, and social factors, such as hormonal changes and various stressors [5, 17, 57]. Therefore, depression may serve as a mediator of the association between gender and LBP, as women are more likely to develop depression, which then increases their risk of experiencing LBP.

Our study had both strengths and limitations. Our study’s primary strength was the utilization of a national representative sample, which would allow generalization of the study’s findings. Furthermore, the large sample size enabled examination of the association between LBP and its determinants with a better power of detection. However, the interpretation of the results needs caution, given the study’s cross-sectional design, which only allows for inferential interpretation than causation. Furthermore, since certain variables such as a personal history of depression and low back pain were self-reported, there is a possibility of recall bias. Because we relied on secondary data for this analysis, we were unable to remove participants who had previously encountered lower back pain from other causes due to non-availability of data. Including individuals who had experienced a significant fall or car accident resulting in lower back pain (LBP), regardless of other underlying factors, could introduce a degree of uncertainty to the study’s conclusions. Even though our study has some limitations, it is important to highlight that the results remain adequately significant. These limitations, although worth mentioning, do not reduce the overall strength and importance of the findings. The significance of the results may be attributed to the sample size being large and representative and a robust study methodology, which emphasizes their importance and validity.

## Conclusion

The results of our study showed that being female and aging were associated with a higher likelihood of experiencing symptoms of low back pain. Additionally, we noted that factors related to behavior, such as body mass index (BMI), significantly predicted the occurrence of low back pain, particularly in adults. Importantly, depression had a potential to mediate the association between gender and low back pain. By understanding that depression serves as a mediator between gender and low back pain, we may pinpoint and address other factors and mechanisms beyond gender and social demographics that play a role in the initiation and exacerbation of low back pain. Moreover, investigating the risk factors associated with low back pain provide an opportunity to increase awareness of the condition on a societal level, which may help reduce its burden. Additionally, public health policymakers and other medical professionals should take these factors into account when developing preventive and treatment programs. Future longitudinal studies are necessary to improve our understanding of the causal relationship between low back pain and its determinants.

## Data Availability

The datasets produced and examined in this study cannot be accessed by the general public. To obtain access to the data, one must apply through the appropriate channels, thus the Türkiye Statistical Institute. However, the corresponding author may provide the datasets upon a reasonable request.
